# Association Between Autoimmune Diseases and Spontaneous Cervicocranial Arterial Dissection

**DOI:** 10.3389/fimmu.2021.820039

**Published:** 2022-01-21

**Authors:** Hao Li, Pu Song, Wei Yang, Le Yang, Shanshan Diao, Shicun Huang, Yiqing Wang, Xingshun Xu, Yi Yang

**Affiliations:** ^1^ Departments of Neurology, The First Affiliated Hospital of Soochow University, Suzhou, China; ^2^ School of Public Health, Fujian Medical University, Fuzhou, China; ^3^ The Institute of Neuroscience, Soochow University, Suzhou, China

**Keywords:** cervicocranial arterial dissection, autoimmune, inflammatory autoimmune disease, inflammatory, neurological disease

## Abstract

A series of biopsies and reports showed autoimmune diseases might be involved in the process of local inflammation related to spontaneous cervicocranial arterial dissection (SCCAD) occurrence. This retrospective case-control study examined the association between SCCADs and autoimmune diseases in patients and control subjects from 2014 to 2020. SCCAD patients and age/sex-matched control subjects were recruited, and clinical data were collected. SCCAD was confirmed by digital subtraction angiography or high-resolution magnetic resonance imaging. The study included 215 SCCAD patients and 430 control subjects. Totally, 135 (62.8%) of the 215 cases were found SCCAD in the anterior circulation, 26 (12.0%) patients involved multiple vessels. Autoimmune disease occurred in 27 (12.6%) cases with SCCAD and 4 (0.9%) control subjects (p<0.001). A conditional multivariable logistic regression model was used to calculate the odds ratio for SCCAD among patients with a history of autoimmune disease, adjusting for hypertension, diabetes, hyperlipidemia, and smoking. After adjustment, autoimmune diseases were associated with SCCAD (p<0.001). After sub-analysis by a similar modeling strategy, significant associations were still observed in different subgroups, such as female group and male group as well as intramural hematoma (IMH) group and Non-IHM group. The association of SCCAD with autoimmune disease suggested that autoimmune mechanisms may be involved in some etiologies of SCCAD.

## Introduction

With imaging technique development, spontaneous cervicocranial arterial dissection (SCCAD) is increasingly diagnosed and becomes a common cause for young adult stroke (1, 2). In a previous study, SCCAD was found to tend to affect multiple arterial segments and develop simultaneously in intracranial and extracranial vessels (2). Although etiologies and pathogenesis of SCCAD have not been fully clarified, a series of biopsies and case reports show that inflammatory alterations and autoimmune diseases might be related to the cascade events leading to SCCAD (3–5). A recent case-control study had also shown that anti-thyroid autoimmunity might be involved in the process of local inflammation related to SCCAD occurrence (6). However, these were not consistent with other studies (7, 8). Due to the limited data, it is unclear whether autoimmune diseases are associated with SCCAD or just co-incident with SCCAD. In this study, we examined the association between SCCAD and autoimmune diseases.

## Materials and Methods

### Study Population

Patients with SCCAD in the First Affiliated Hospital of Soochow University from April 2014 to October 2020 were selected. Controls were randomly selected from individuals that resided in Suzhou City and attended the annual physical examination in our hospital and matched the patients with age and gender. All patients and controls underwent cervical cerebrovascular ultrasonography to screen SCCAD. Suspected SCCAD was further confirmed by digital subtraction angiography (DSA) or high-resolution magnetic resonance imaging (HR-MRI). Key pathognomonic radiological findings of CCAD were confirmed, including intimal flap, intramural hematoma (IMH), and double lumen. Exclusion criteria were as follows: 1) patients with definite traumatic CCAD; 2) patients had a history of iatrogenic cervicocranial artery injury or craniocervical vascular surgery within 6 months; 3) patients with Stanford type A aortic dissection involving the carotid artery; 4) patients with localized subclavian artery dissection.

### Autoimmune Diseases Detection

Autoimmune diseases were diagnosed according to the International Classification of Diseases-9th Revision (ICD-9) and were classified as autoinflammatory diseases and classic autoimmune diseases (9) (**Table 1** and its notes). Autoimmune disease history was defined as the definite diagnosis that should be made before or within one month after the SCCAD events. The diagnosis of autoimmune diseases was based on a clinical interview, physical examination, and a medical record system that contained medical information including medical histories, diagnostic tests, diagnosis, and treatments for individuals who resided in Suzhou city and received health care within the system.

**Table 1:** Characteristics and autoimmune diseases of all 215 cases with SCCAD and their control subjects of first SCAD event.

	SCCAD cases (n=215)	Control subjects (n=430)	*p*-value
Age in years, median (IQR)	48 (38, 58)	49 (38, 58)	0.969
Male, n (%)	134 (62.3)	268 (62.3)	1.000
Smoking, n (%)	25 (11.6)	51 (11.9)	0.931
Hypertension, n (%)	85 (39.5)	166 (38.6)	0.819
Diabetes, n (%)	28 (13.0)	53 (12.3)	0.801
Hyperlipidemia, n (%)	49 (22.8)	100 (23.3)	0.895
Anterior circulation, n (%)	135 (62.8)	N/A	N/A
Intramural hematoma, n (%)	85 (39.5)	N/A	N/A
Multiple vessels involved	26 (12.0)	N/A	N/A
Cerebral infarction	142 (66.0)	N/A	N/A
With at least 1 autoimmune disease, n (%)	27 (12.6)	4 (0.9)	<0.001
Disease*			
Systemic lupus erythematosus, n (%)	2 (0.9)	0 (0.0)	0.111
Pernicious anemia, n (%)	1 (0.5)	0 (0.0)	0.157
Takayasu arteritis, n (%)	3 (1.4)	0 (0.0)	0.014
Sjőgren’s syndrome, n (%)	1 (0.5)	0 (0.0)	0.157
Antiphospholipid syndrome, n (%)	4 (1.9)	0 (0.0)	0.012
Rheumatoid arthritis, n (%)	0 (0.0)	1 (0.2)	1.000
Ankylosing spondylitis, n (%)	1 (0.5)	0 (0.0)	0.157
Vasculitis, n (%)	1 (0.5)	1 (0.2)	1.000
Psoriasis or psoriatic arthritis, n (%)	0 (0.0)	2 (0.4)	0.045
Myasthenia gravis, n (%)	1 (0.5)	0 (0.0)	0.333
Autoimmune thyroid disease, n (%)	15 (7.0)	0 (0.0)	<0.001
Hepatitis, autoimmune, n (%)	1 (0.5)	0 (0.0)	0.333

*Participants could have >1 disease. None of Participants had Addison’s disease, Celiac disease, Crohn’s disease, Dermatomyositis/polymyositis, Guillain-Barre syndrome, Multiple sclerosis,Polymyalgia rheumatica, Primary biliary cirrhosis, Systemic sclerosis, Type 1 diabetes, Ulcerative colitis, Uveitis, or Vitiligo.

NA, Not applicable.

### Clinical Information Collection

For all subjects, we collected baseline data including cerebral vascular risk factors such as hypertension and diabetes; affected vessel, and vessel states (occlusion or stenosis). Antinuclear antibodies, antineutrophil cytoplasmic antibodies, anticardiolipin antibodies, anticitrullinated protein antibodies, tumor-associated antigens (including tumor specific growth factor with chemiluminescence assay), thyroid stimulating hormone, and anti-thyroid stimulating hormone receptor antibodies were detected in some patients and controls to clarify the diagnosis.

### Statistical Analysis

Conditional multivariable logistic regression analysis provided the adjusted odds ratio (OR) with a 95% confidence interval (CI) for odds of SCCAD in the presence of at least 1 autoimmune disease after adjusting for other variables selected from univariate analyses. Furthermore, participants were stratified and analyzed by a similar modeling strategy as for the main model. Statistical analysis was performed in SPSS 25.0. *P*<0.05 was considered statistically significant.

## Results

### SCCAD Cases and Characteristics

In this study, 215 patients with SCCAD and 430 control subjects were included. For patients with SCCAD, their median age was 48 (38, 58) years, 134 (62.3%) patients were male. For control subjects, their median age was 49 (38, 58) years and 268 (62.3%) patients were male. There was no difference in smoking, hypertension, diabetes, and hyperlipidemia between the two groups (**Table 1**). More importantly, 27 (12.6%) cases in SCCAD patients and 4 (0.9%) cases in control subjects had at least 1 autoimmune disease (**Table 1**). There was a significant increase in the percentage of patients with the autoimmune disease in the SCCAD group compared with controls (*p*<0.001). In addition, an increase in tumor specific growth factor (TSGF) level was observed in 72.6% SCCAD patients (45/62 patients). Five SCCAD patients had isolated anti-nuclear antibodies without clinical manifestation.

### Autoimmune Disease and the Risk of SCCAD

We further analyzed the composition of autoimmune diseases in SCCAD patients and control subjects. Compared with control subjects, autoimmune thyroid disease (AITD), antiphospholipid syndrome, and Takayasu arteritis were more common in SCCAD patients (*p*<0.05, **Table 1**). Other autoimmune diseases occurred in both groups with a low frequency (*p*>0.05, **Table 1**). To demonstrate the association between autoimmune diseases and SCCAD, conditional univariate and multivariable logistic regression analyses were performed. After adjustment for hypertension, diabetes, hyperlipidemia and smoking, having autoimmune diseases was significantly associated with increased odds of SCCAD (OR: 2.873; 95% CI: 1.914-4.311; *p<*0.001, **Table 2**).

**Table 2:** Conditional multivariable logistic regression analysis showed having an autoimmune disease was associated with increased odds of SCCAD.

Factor	Univariate logistic regression analysis		Multivariable logistic regression analysis	
	OR (95% CI)	*p*-value	OR (95% CI)	*p*-value
With at least 1 autoimmune disease	2.845 (1.9-4.258)	<0.001	2.873 (1.914-4.311)	<0.001
Smoking	0.985 (0.649-1.495)	0.944	0.955 (0.625-1.461)	0.834
Hypertension	1.026 (0.781-1.349)	0.852	0.984 (0.736-1.316)	0.915
Diabetes	1.043 (0.701-1.551)	0.837	1.101 (0.729-1.662)	0.648
Hyperlipidemia	0.983 (0.714-1.351)	0.914	0.987 (0.715-1.364)	0.938

To support our hypothesis, we performed a sub-analysis including patients with or without intramural hematoma (IMH, a pathognomonic marker of SCCAD) (10–12). Interestingly, 85 (39.5%) cases with SCCAD had IMH; among them, 15 (17.6%) patients had at least one autoimmune disease; 130 (60.5%) cases of SCCAD had no IMH, and 12 (9.2%) patients had at least one autoimmune disease. There was no difference in the case ratio of autoimmune diseases between the IMH group and the Non-IMH group (**Table 3**; *p*>0.05). After the analysis with a similar modeling strategy, significant associations between were observed in both sub-groups (IMH group: OR: 3.146; 95% CI: 1.76-5.621; p<0.001; non-IMH group: OR: 2.523; 95% CI: 1.389-4.582; p=0.002; **Tables 4**, **5**), indicating that autoimmune diseases were associated with SCCAD, but not with IMH.

**Table 3:** Characteristics in patients with and without IMH.

	IMH group (n=85)	non-IMH group (n=130)	*p*-value
Age in years, median (IQR)	47.56 ± 11.78	49.04 ± 13.83	0.419
Male, n (%)	56 (65.9)	78 (60.0)	0.384
Smoking, n (%)	12 (14.1)	13 (10.0)	0.357
Hypertension, n (%)	40 (47.1)	45 (34.6)	0.068
Diabetes, n (%)	9 (10.6)	19 (14.6)	0.391
Hyperlipidemia, n (%)	24 (28.2)	25 (19.2)	0.124
With at least 1 autoimmune disease, n (%)	15 (17.6)	12 (9.2)	0.069

**Table 4:** Conditional multivariable logistic regression analysis in SCCAD patients with IMH.

Factor	Univariate logistic regression analysis		Multivariable logistic regression analysis	
	OR (95% CI)	p-value	OR (95% CI)	*p*-value
With at least 1 autoimmune disease	3.201 (1.833-5.591)	<0.001	3.146 (1.76-5.621)	<0.001
Smoking	1.333 (0.871-2.041)	0.186	1.243 (0.798-1.938)	0.336
Hypertension	0.96 (0.481-1.916)	0.908	1.065 (0.526-2.157)	0.86
Diabetes	0.962 (0.6-1.543)	0.873	0.987 (0.608-1.601)	0.958
Hyperlipidemia	1.068 (0.58-1.968)	0.832	0.906 (0.484-1.694)	0.756

**Table 5:** Conditional multivariable logistic regression analysis in SCCAD patients without IMH.

Factor	Univariate logistic regression analysis		Multivariable logistic regression analysis	
	OR (95% CI)	p-value	OR (95% CI)	*p*-value
With at least 1 autoimmune disease	2.542 (1.404,4.604)	0.002	2.523 (1.389,4.582)	0.002
Smoking	0.921 (0.519,1.633)	0.777	1.003 (0.559,1.801)	0.991
Hypertension	0.837 (0.582,1.203)	0.336	0.79 (0.533,1.171)	0.241
Diabetes	1.173 (0.736,1.872)	0.502	1.264 (0.77,2.074)	0.354
Hyperlipidemia	0.967 (0.621,1.506)	0.881	0.965 (0.617,1.511)	0.878

Previous studies showed that autoimmune diseases are more prevalent in females (13); however, this retrospective cohort study showed predominantly male with SCCAD. Therefore, we analyzed the percentage of SCCAD patients with autoimmune diseases by gender. We found that 14 (17.3%) female SCCAD patients had autoimmune diseases, but only 13 (9.7%) male SCCAD patients had autoimmune diseases, indicating that autoimmune diseases were still more prevalent in female SCCAD patients than in male SCCAD patients. To further clarify the impact of gender on the association between autoimmune diseases and SCCAD, the patients and control subjects were divided into two groups by gender. After analyzing by a similar modeling strategy as for the main model, significant associations were still observed in two sub-groups, especially in female SCCAD patients with high OR value (female: OR: 3.254; 95% CI: 1.822-5.809; *p<*0.001; male: OR: 2.677; 95% CI: 1.491-4.806; *p=*0.001).

## Discussion

SCCAD is an infrequent but potentially devastating cause of stroke; its pathophysiologic mechanisms remain somewhat unclear (1). Although some case studies had discussed the possible relationship between SCCAD and autoimmune diseases, available data were sparse and inconsistent (3, 6–8, 14). In our cohort study on SCCAD patients, a higher ratio of autoimmune diseases was observed. The autoimmune disease was significantly associated with SCCAD.

The pathologic and imaging evidence of inflammatory infiltrates in the wall of intracranial dissected arteries suggest that local inflammatory alterations might be a crucial step for SCCAD (6). SCCAD with mural hematoma is more frequently associated with the presence of periarterial edema compared with traumatic mural hematoma, which supported the concept of underlying arterial inflammation in SCCAD (15). Inflammatory markers were elevated in stroke patients with SCCAD and correlated with clinical prognosis (16). In addition, two reports also showed that inflammation in the arterial wall was associated with SCCAD and aspirin/steroids treatment resulted in dramatic improvement of both clinical condition and magnetic resonance imaging brain lesions (3, 17). Failure to demonstrate a specific infection agent led to a hypothesis that the activation of specific immune-mediated mechanisms rather than a specific infective agent may be responsible for a local inflammatory alteration linked to SCCAD.

Meanwhile, SCCAD biopsy specimens demonstrated the presence of a generalized arteriopathy that leads to the impairment of the stability of arterial walls (4). In a previous study on aneurysm, proinflammatory cytokines could be activated by immune mechanisms and induce the proteolytic process for the degradation of the extracellular matrix proteins (18). It is difficult to determine whether the inflammatory reaction is the cause or the consequence of histopathologic changes in SCCAD. However, immune-related inflammatory infiltration was absent in traumatic dissection (15, 19). In some case reports, these arterial anomalies disappeared rapidly after immunosuppressive therapy, which also supports the link between autoimmunity and dissection (3, 17, 20).

Most autoimmune diseases are more prevalent in females than in males (13). Symptom severity, disease course, response to therapy, and overall survival may also differ between males and females with autoimmune diseases (13). Sex hormones have a crucial role in this gender bias because estrogens are potent stimulators of autoimmunity (13). However, it appears to be a slight gender predisposition favoring males in population-based studies of SCCAD, which were similar to our results, arguing against an estrogen effect (1). This may be related to the protective influence of estrogen on the cerebral vasculature. Meanwhile, our results showed that the proportion of female SCCAD patients with autoimmune diseases was much higher than that of male patients, supporting that autoimmune diseases are more prevalent in females.

In this study, we demonstrated the close association between SCCAD and some autoimmune diseases like AITD, antiphospholipid syndrome, and Takayasu arteritis, but not other autoimmune diseases. So far, current studies have not yet elucidated why certain organs or vessels become the targets of immune injury in autoimmune diseases while others are spared. This may be explained by the presence of specific autoimmune antibodies that cross-react with proteins present in cervicocranial arteries in different autoimmune diseases, which needs more investigation.

The main strengths of our study are the inclusion of a relatively large cohort of verified SCCAD patients; however, our data should be interpreted with some caution. There may have retrospective bias inherent, and the results may not generalize outside of the studied geographic location due to the variety of autoimmune diseases by latitude.

In our study, autoimmune disease was associated with SCCAD, suggesting disorders of immunity might have a role in the mechanism of local inflammatory alterations leading to SCCAD.

## Data Availability Statement

The raw data supporting the conclusions of this article will be made available by the authors, without undue reservation.

## Ethics Statement

The studies involving human participants were reviewed and approved by The Institutional Review Board of the First Affiliated Hospital of Soochow University. The patients/participants provided their written informed consent to participate in this study.

## Author Contributions

Conception and design of the study: XX and YY. Data acquisition and analysis of data: HL, PS, WY, LY, SD, SH, YW, and YY. Drafting the manuscript: HL, PS, XX, and YY. All authors commented on previous versions of the manuscript. All authors contributed to the article and approved the submitted version.

## Funding

This work was supported by National Science Foundation of China (82071511), Shandong Provincial Natural Science Foundation (ZR2019ZD32), and the Priority Academic Program Development of Jiangsu Higher Education Institutions (20KJB320021).

## Conflict of Interest

The authors declare that the research was conducted in the absence of any commercial or financial relationships that could be construed as a potential conflict of interest.

## Publisher’s Note

All claims expressed in this article are solely those of the authors and do not necessarily represent those of their affiliated organizations, or those of the publisher, the editors and the reviewers. Any product that may be evaluated in this article, or claim that may be made by its manufacturer, is not guaranteed or endorsed by the publisher.
